# Longitudinal biomarkers and kidney disease progression after acute kidney injury

**DOI:** 10.1172/jci.insight.167731

**Published:** 2023-05-08

**Authors:** Yumeng Wen, Leyuan Xu, Isabel Melchinger, Heather Thiessen-Philbrook, Dennis G. Moledina, Steven G. Coca, Chi-yuan Hsu, Alan S. Go, Kathleen D. Liu, Edward D. Siew, T. Alp Ikizler, Vernon M. Chinchilli, James S. Kaufman, Paul L. Kimmel, Jonathan Himmelfarb, Lloyd G. Cantley, Chirag R. Parikh, the ASSESS-AKI Consortium

**Affiliations:** 1 Division of Nephrology, Department of Medicine, Johns Hopkins University School of Medicine, Baltimore, Maryland, USA.; 2 Section of Nephrology, Department of Medicine, Yale School of Medicine, New Haven, Connecticut, USA.; 3 Division of Nephrology, Department of Medicine, Icahn School of Medicine at Mount Sinai, New York, New York, USA.; 4 Division of Nephrology, University of California, San Francisco, San Francisco, California, USA.; 5 Kaiser Permanente Division of Research, Oakland, California, USA.; 6 Division of Nephrology, Vanderbilt University, Nashville, Tennessee, USA.; 7 Division of Nephrology, Pennsylvania State University College of Medicine, Hershey, Pennsylvania, USA.; 8 Division of Nephrology, New York University School of Medicine and VA New York Harbor Healthcare System, New York, New York, USA.; 9 National Institute of Diabetes and Digestive and Kidney Diseases (NIDDK), National Institutes of Health (NIH), Bethesda, Maryland, USA.; 10 Division of Nephrology, University of Washington, Seattle, Washington, USA.; 11 The ASSESS-AKI Consortium is detailed in Supplemental Acknowledgments.

**Keywords:** Nephrology, Chronic kidney disease, Diagnostics, Epidemiology

## Abstract

**BACKGROUND:**

Longitudinal investigations of murine acute kidney injury (AKI) suggest that injury and inflammation may persist long after the initial insult. However, the evolution of these processes and their prognostic values are unknown in patients with AKI.

**METHODS:**

In a prospective cohort of 656 participants hospitalized with AKI, we measured 7 urine and 2 plasma biomarkers of kidney injury, inflammation, and tubular health at multiple time points from the diagnosis to 12 months after AKI. We used linear mixed-effect models to estimate biomarker changes over time, and we used Cox proportional hazard regressions to determine their associations with a composite outcome of chronic kidney disease (CKD) incidence and progression. We compared the gene expression kinetics of biomarkers in murine models of repair and atrophy after ischemic reperfusion injury (IRI).

**RESULTS:**

After 4.3 years, 106 and 52 participants developed incident CKD and CKD progression, respectively. Each SD increase in the change of urine KIM-1, MCP-1, and plasma TNFR1 from baseline to 12 months was associated with 2- to 3-fold increased risk for CKD, while the increase in urine uromodulin was associated with 40% reduced risk for CKD. The trajectories of these biological processes were associated with progression to kidney atrophy in mice after IRI.

**CONCLUSION:**

Sustained tissue injury and inflammation, and slower restoration of tubular health, are associated with higher risk of kidney disease progression. Further investigation into these ongoing biological processes may help researchers understand and prevent the AKI-to-CKD transition.

**FUNDING:**

NIH and NIDDK (grants U01DK082223, U01DK082185, U01DK082192, U01DK082183, R01DK098233, R01DK101507, R01DK114014, K23DK100468, R03DK111881, K01DK120783, and R01DK093771).

## Graphical abstract

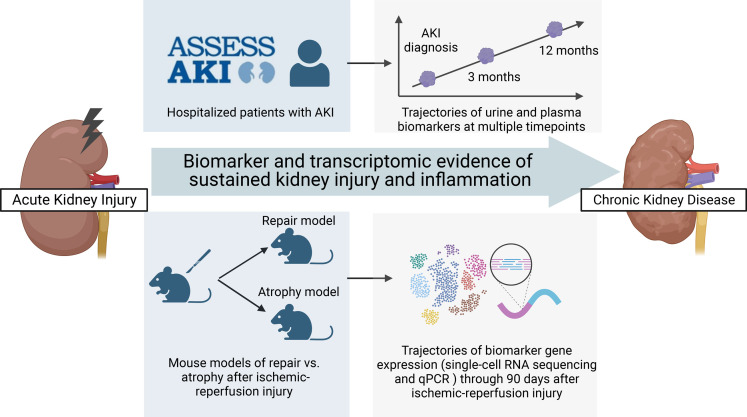

## Introduction

Acute kidney injury (AKI) represents a sudden decline in kidney function and is typically caused by ischemic, toxic, or inflammatory injury to the renal tubules (1). AKI complicates the course of illness in approximately 20% of hospitalized patients (2). Once viewed as a self-limited and reversible condition, AKI is now increasingly recognized as a significant risk factor for long-term cardiovascular and kidney complications (3, 4). Hospitalized individuals with AKI have 3- to 8-fold increased risks for developing incident chronic kidney disease (CKD) or progression of preexisting CKD, and they have 3-fold increased risks for developing end-stage kidney disease (5, 6). On the other hand, CKD has affected more than 850 million individuals worldwide, has become the eighth leading cause of mortality in the United States, and is associated with rising health care costs (7). AKI incidence in the hospital is continuously rising; therefore, understanding how and why AKI predisposes to the development of CKD is important in improving clinical care and in providing mechanistic insights for the development of therapies to mitigate this long-term complication.

Emerging evidence from longitudinal studies of mouse models of AKI suggest that the tissue recovery course of AKI may last beyond 4–6 weeks after initial injury, especially in cases of severe injury (8–11). In mice after ischemic reperfusion injury (IRI), although most injured tubular cells are able to return to the healthy state in 7–10 days, a subgroup of injured tubular cells diverge to a senescent phase, activate immune cells and fibroblasts, and result in interstitial fibrosis and long-term kidney function loss (8, 10–12). This maladaptive process emerges approximately 2 days after IRI, worsens in the first 2 weeks, and can persist months after IRI despite significant improvement in the glomerular filtration rate (GFR) (8). Recent transcriptomic studies of human AKI demonstrated similar maladaptive response at the tubular cell level (13). However, due to the cross-sectional study designs, it is unclear how the injury and inflammation processes evolve over time in patients with AKI and whether the interrogation of kidney injury and inflammation processes will help with the phenotyping of AKI by discerning maladaptive versus adaptive repair and by providing prognostication for kidney disease progression after AKI. Previous studies used cross-sectional measurements of biomarkers to evaluate kidney injury, inflammation, and tubular health in patients with AKI (14). However, biomarker measurements at a singular time point may not sufficiently capture the evolution of these biological processes over time. In this study, we measured biomarkers at multiple time points up to a year after AKI to determine the associations of longitudinal changes in these biomarkers with progression of kidney disease after AKI. We also aimed to gain insights into whether the trajectories of kidney injury and inflammation in patients are aligned with tubular cell maladaptive responses and fibrosis in mouse models of IRI followed by kidney atrophy compared with repair. We hypothesized that sustained kidney injury and inflammation, and slower restoration of tubular health after AKI diagnosis, were associated with higher risks of developing CKD in hospitalized patients and with kidney atrophy after IRI in mice.

## Results

### Clinical characteristics of participants with AKI.

**Figure 1:**
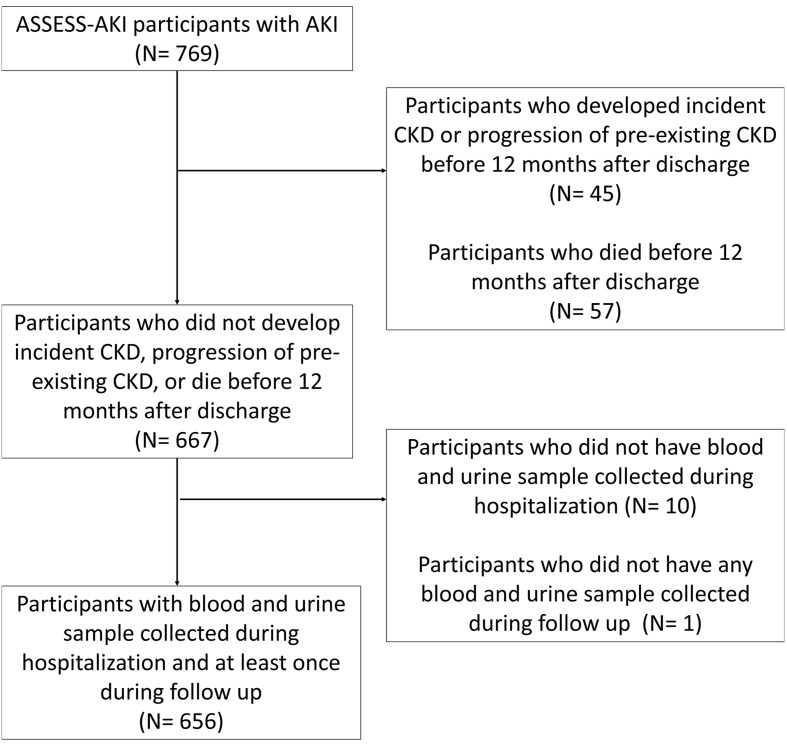
Flow chart for the inclusion of study participants in ASSESS-AKI cohort.

Among 769 participants with AKI enrolled in the Assessment, Serial Evaluation, and Subsequent Sequelae of Acute Kidney Injury (ASSESS-AKI) study, we excluded 45 participants who developed incident CKD or progression of preexisting CKD prior to their 12 months of study visits because they already developed the outcome before the start of the observation. We also excluded 57 participants who did not develop the CKD outcome but died before their 12-month study visits, because the study outcome could no longer be observed in these participants. Among the remaining 667 participants, 656 participants had blood and urine sample collected during hospitalization (i.e., within 48–96 hours of AKI diagnosis), had at least 1 blood and urine sample collected at their 3- and 12-month visits, and were included in the study (Figure 1). The median (IQR) age of these participants was 65 years (56–73 years). One hundred (15.2%) participants self-identified as Black, and 15 (2.3%) participants self-identified as having Hispanic ethnicity (Table 1). The median (IQR) baseline estimated GFR (eGFR) was 66.3 mL/min/1.73 m^2^ (49.4–85.2 mL/min/1.73 m^2^), and 259 (39.5%) participants had baseline CKD prior to hospitalization. After 4.3 years (median) of follow-up from hospitalization, 158 (24.1%) participants developed a composite CKD outcome of incident CKD and CKD progression, including 106 of 397 (26.7%) participants with incident CKD and 52 of 259 (20.1%) participants with progression of preexisting CKD.

### Biomarker trajectories in the first 12 months after hospitalization with AKI.

We used linear mixed effect models to estimate the longitudinal changes (i.e., slopes) in biomarkers of kidney injury (urine albumin, kidney injury molecule-1 [KIM-1], and neutrophil gelatinase–associated lipocalin [NGAL]), inflammation (urine IL-18, monocyte chemoattractant protein-1 [MCP-1], chitinase 3-like 1 [YKL-40], and plasma soluble TNF receptor 1 and 2 [TNFR1 and TNFR2]), and tubular health (urine uromodulin [UMOD]) from the hospitalization time point and over the first year of follow-up. We found that, while most biomarkers decreased over time on average (median), there was a large variability among patients, as represented by the wide ranges (IQR) of biomarker slopes (Table 2). Compared with participants who did not develop the composite CKD outcome, participants developing the outcome had increasing kidney injury and inflammation biomarkers as well as decreasing tubular health biomarkers. These differences were persistent after excluding 95 participants with recurrent AKI between hospitalization and 12-month follow-up (Supplemental Table 1; supplemental material available online with this article; https://doi.org/10.1172/jci.insight.167731DS1).

### Biomarker slopes and CKD outcomes after AKI.

**Figure 2:**
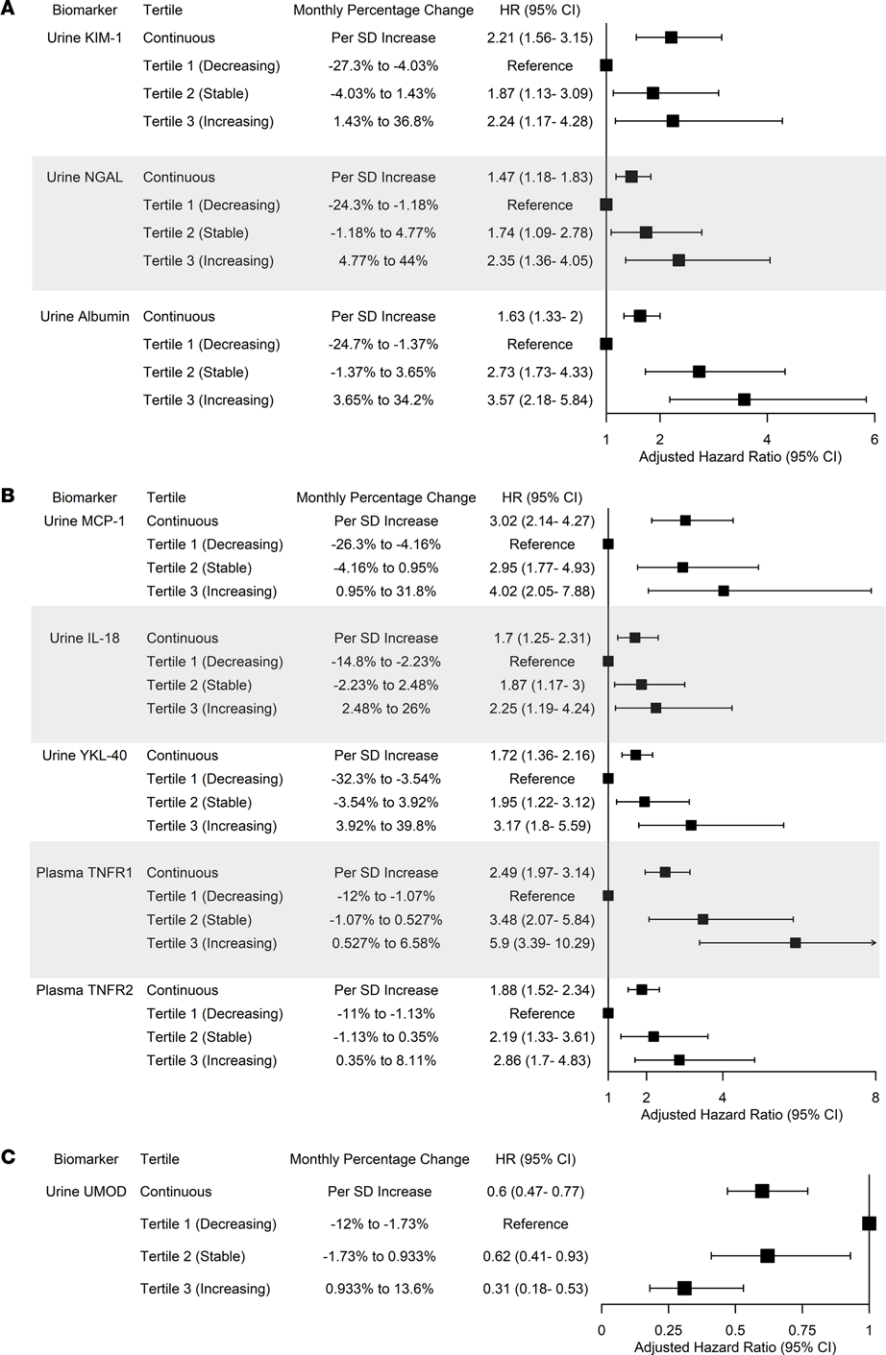
Associations between slopes of biomarkers of kidney injury, inflammation, and tubular health with the composite outcome of incident CKD and CKD progression. (**A**–**C**) Association between biomarkers of kidney injury (**A**), inflammation (**B**), and tubular health (**C**) with the composite CKD outcome in 656 participants with AKI in the ASSESS-AKI cohort. Cox proportional hazard regression models were adjusted for biomarker at hospitalization, age, sex, race, Hispanic ethnicity, hypertension, diabetes, atherosclerotic disease, congestive heart failure, smoking status, baseline eGFR, albuminuria at hospitalization, urine creatinine at hospitalization, and the slope of urine creatinine from hospitalization to 12 months after discharge. Follow-up started at 12 months after hospitalization, and participants who died were censored. Median follow-up duration was 4.3 years.

We subsequently determined whether the longitudinal changes in biomarkers of kidney injury, inflammation, and tubular health after AKI diagnosis were associated with kidney disease progression after AKI. For participants with similar biomarker levels during hospitalization, each SD increase in the biomarker slopes of kidney injury and inflammation was associated with higher risk of developing the CKD outcome, while each SD increase in the biomarker slope of tubular health (i.e., urine UMOD) was associated with lower risk of the CKD outcome (Table 3 and Supplemental Table 3). The associations were persistent after adjusting for demographic characteristics, comorbidities, baseline eGFR, and urinary album excretion during hospitalization with AKI (Figure 2). In the fully adjusted models, each SD increase in the slopes for urine KIM-1, MCP-1, and TNFR1 was associated with 2- to 3-fold higher risks of developing the CKD outcome. These associations were consistent after excluding 95 participants who developed recurrent AKI between hospitalization and 12-month follow-up visits (Supplemental Figure 1) and when evaluating biomarker slopes as tertiles (Figure 2). In addition, participants in the higher tertiles of biomarker slopes experienced faster kidney function decline (Supplemental Figure 2). When evaluating these associations in subgroups of participants with and without preexisting CKD, we found that having biomarker evidence of persistent kidney injury, inflammation, and lack of restoration of tubular health was associated with incident CKD in those without preexisting CKD as well as with CKD progression in those with preexisting CKD (Supplemental Figure 3, A and B).

**Figure 3:**
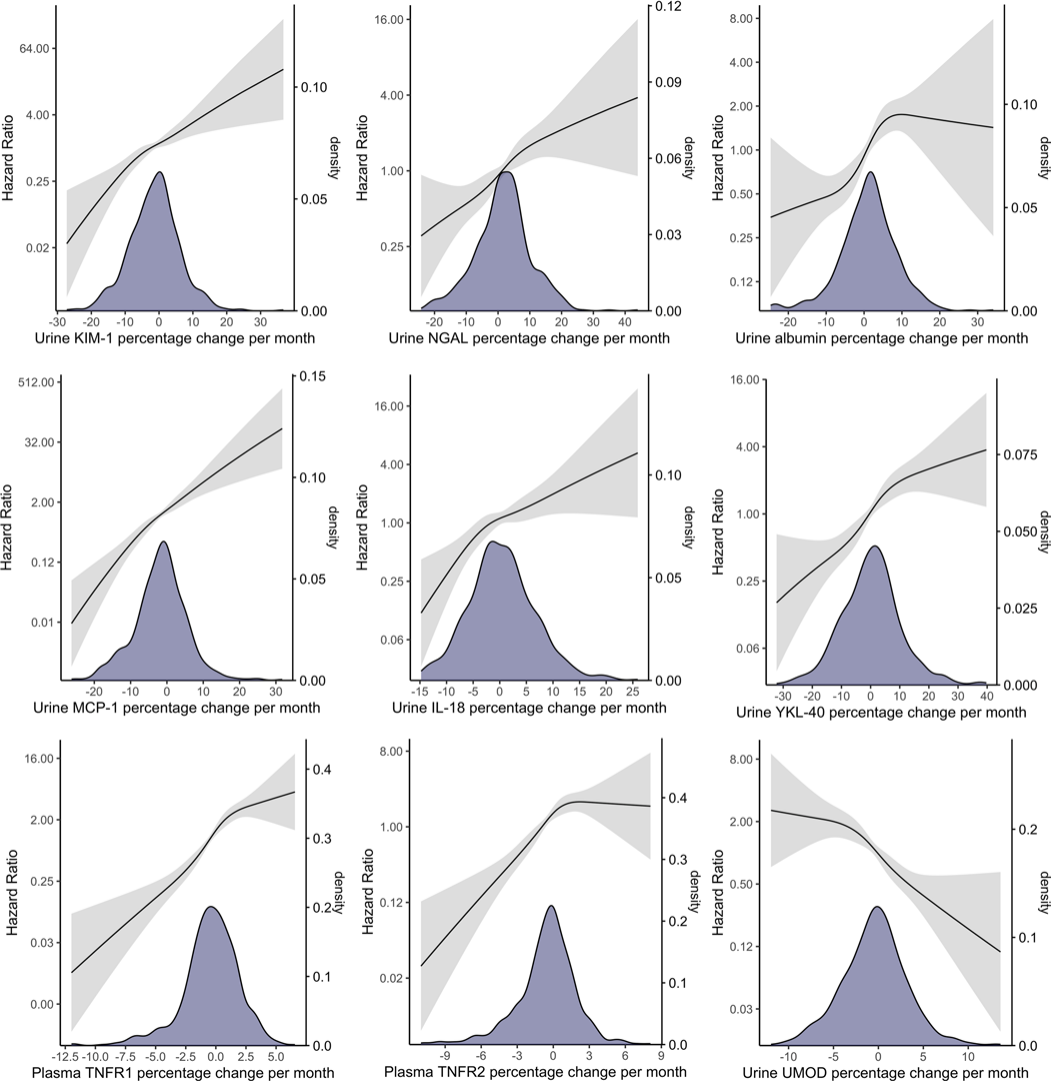
Distribution of monthly biomarker changes and the associations between biomarker slopes using restricted cubic splines with the composite CKD outcome in 656 participants with AKI. Distribution of biomarker slopes were visualized using kernel density plot to aid the interpretation of the confidence in hazard ratio estimates. Cox proportional hazard regression models were adjusted for biomarker at hospitalization, age, sex, race, Hispanic ethnicity, hypertension, diabetes, atherosclerotic disease, congestive heart failure, smoking status, baseline eGFR, albuminuria at hospitalization, urine creatinine at hospitalization, and the slope of urine creatinine from hospitalization to 12 months after discharge.

Because the associations between biomarker slopes and CKD in participants with resolving injury (i.e., negative biomarker slope) and participants with worsening injury (i.e., positive biomarker slope) may be qualitatively different, we explored the nonlinear relationships using restrictive cubic splines of the biomarker slopes with the CKD outcome. We observed steadily increasing risks of CKD as biomarker slopes increase for most biomarkers (Figure 3). Compared with participants with mean biomarker slopes that were approximately zero, those with negative biomarker slopes had lower risks of CKD, and the risks further decreased as the slopes became smaller (i.e., more negative). On the other hand, those with positive biomarker slopes had higher risks of CKD compared with participants with zero or negative biomarker slopes, and the risks increased as the slopes became larger (i.e., more positive). These findings suggest that: (a) for participants with resolving kidney injury and inflammation (i.e., decreasing, negative biomarker slopes), rapid resolution (i.e., lower negative slope) was associated with lower risks of CKD and slower resolution was associated with higher risks of CKD; (b) for participants with worsening kidney injury and inflammation (i.e., increasing, positive biomarker slopes), their risks of developing the CKD outcome were higher than those with resolving kidney injury and inflammation; and (c) higher rates of worsening injury and inflammation (i.e., larger positive slope) were associated with higher risks of CKD. For participants with urine albumin increasing more than 10% per month and for participants with an increasing plasma TNFR2 (positive slope), their risks of developing a CKD outcome did not increase further.

Although the mixed effect models account for the absence of sample collection at 3 months and 12 months after hospitalization, they assume the changes of biomarkers after AKI are linear from hospitalization with AKI to 12 months after hospitalization. Therefore, we conducted a sensitivity analysis by modeling biomarker slopes from hospitalization to 3 months, and from 3 months to 12 months after hospitalization as 2 exposures, in participants who had urine and blood samples collected at all 3 time points. Among the 656 AKI participants, 581 and 541 had urine and blood samples collected, respectively, at all 3 time points. The increase in the slopes of injury and inflammation biomarkers, and the decrease in the slope of tubular health biomarker at both time intervals, were independently associated with higher risks of the composite CKD outcome, and the associations were stronger for biomarker slopes in the first 3 months after AKI (Supplemental Figure 4).

### Longitudinal biomarker gene expressions and kidney atrophy in mice after IRI.

**Figure 4:**
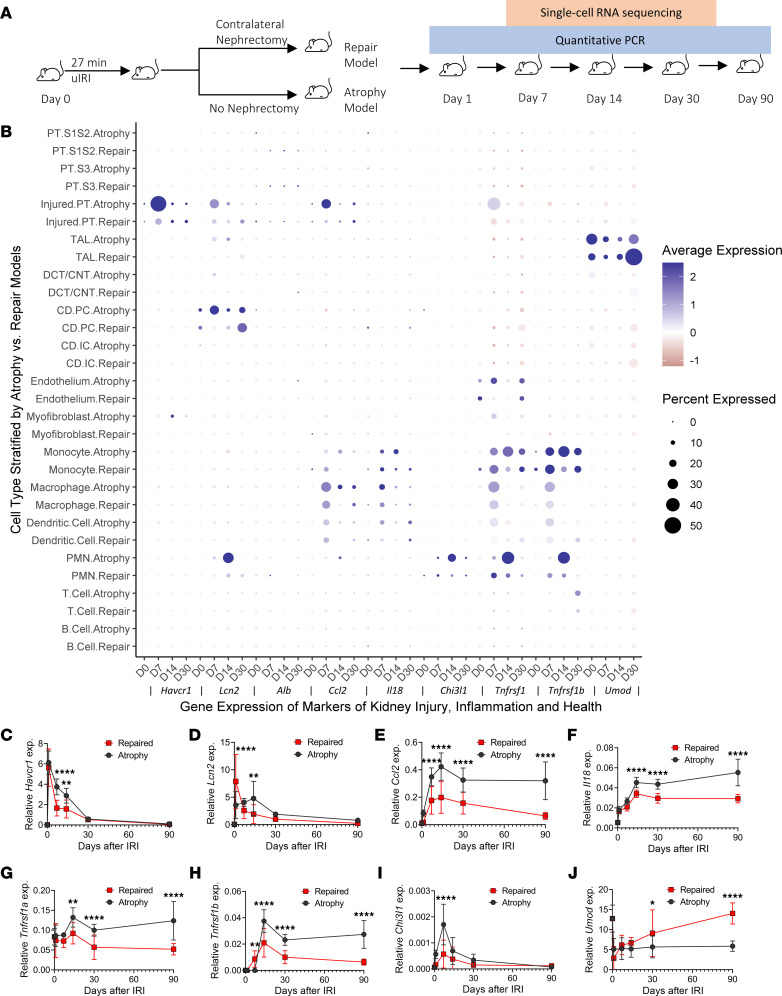
Comparison of the gene expressions of biomarkers of kidney injury, inflammation, and tubular health in mouse models of atrophy and repair after ischemic reperfusion injury. (**A**) Overview of animal experiments. WT mice were subjected to 27 minutes of unilateral IRI with the contralateral kidney intact (atrophy model) or unilateral IRI with contralateral nephrectomy (repair model) followed by single-cell isolation and sequencing (scRNA-Seq and qPCR) at the indicated time points (14). (**B**) Expression of mRNA for biomarkers across 95,343 cells of the indicated identity (*y* axis) in mouse models of repair and atrophy at multiple time points after IRI (*n* = 2 kidneys for control; *n* = 2 kidneys/time point/model for days 7, 14, and 30). (**C**–**J**) qPCR analysis for *Havcr1*, *Lcn2*, *Ccl2*, *Il18*, *Chi3l1*, *Tnfrsf1a*, *Tnfrsf1b*, and *Umod* was performed on whole-kidney RNA harvested 0, 1, 7, 14, 30, and 90 days after injury; *n* = 10 kidneys/time point/model. *P < 0.05, **P < 0.01, ****P < 0.0001 at the indicated time point using 2-way ANOVA followed by Bonferroni post hoc test for subgroup analysis.

We qualitatively compared the gene expression of these biomarkers using single-cell RNA-Seq (scRNA-Seq) of 95,343 kidney cells at multiple time points in mouse models of IRI followed by kidney atrophy versus kidney repair (Figure 4A) (14, 15). Compared with the repair model, kidney atrophy correlated with a higher proportion of injured proximal tubular cells expressing injury markers *Havcr1* (gene encoding KIM-1) and *Lcn2* (gene encoding NGAL), as well as inflammation marker *Ccl2* (gene encoding MCP-1) and *Tnfrsf1a* (gene encoding TNFR1), at 7–14 days after IRI (Figure 4B). Similarly, there were higher proportions of distal tubular cells (thick ascending limb and collecting duct principal cells) expressing *Lcn2*, macrophages expressing *Ccl2*, and polymorphonuclear neutrophils expressing *Lcn2* and *Chi3l1* (gene encoding YKL-40), and there was a lower proportion of thick ascending limb cells expressing tubular health marker *Umod* in the atrophy model at these time points. These differences in gene expression suggest that sustained expression of injury-associated tubular cell genes and the coincident inflammatory cell activation associate with kidney atrophy during AKI-to-CKD transition.

To further explore the associations between gene expression trajectories and fibrosis after IRI, we determined the gene expression kinetics of these markers at the whole kidney mRNA level and compared their longitudinal changes in the atrophy and repair models (Figure 4, C–J, and Supplemental Table 4). For biomarkers of kidney injury, while both models had decreases in *Havcr1* expression 1 day after IRI, the decrease was significantly slower for the atrophy model. In the repair model, we observed a steady decrease in *Lcn2* expression after day 1. In the atrophy model, however, *Lcn2* expression was persistently high in the first 14 days after IRI. In addition, we observed no recovery of the tubular health marker (*Umod*) in the atrophy model after day 7, consistent with failure to resolve tubular injury in this model. Both atrophy and repair models displayed kidney inflammation — i.e., *Il18*, *Tnfrsf1a*, *Tnfrsf1b* (gene encoding TNFR2), and *Ccl2 —* in the first 14 days after IRI. However, the peak expression of these inflammation markers was significantly greater in the atrophy model, with inflammation persisting through day 90 in these atrophied kidneys, whereas the repair model exhibited progressive resolution in the inflammatory process.

## Discussion

In this study, we measured a panel of noninvasive biomarkers reflecting kidney injury, inflammation, and tubular health at multiple time points in hospitalized patients with AKI. We found that patients with biomarker evidence of sustained injury and inflammation, and slower restoration of tubular health, were at higher risk of developing incident CKD and experiencing progression of preexisting CKD. To gain mechanistic insights into these associations, we performed transcriptomic comparison of 2 mouse models of IRI followed by either repair or atrophy. We found that sustained injury and inflammation, and slower restoration of tubular health, were associated with maladaptive repair of the renal tubule and with subsequent kidney fibrosis and atrophy in mouse models (14, 15).

The repair process in the injured renal tubule starts soon after the initial insult in murine models of AKI. Most injured tubular cells are able to undergo redifferentiation and proliferation and return to the healthy state by 7–10 days (16). However, in the instance of severe injury, a subgroup of cells may diverge into a senescent phase and remain undifferentiated. These failed-to-repair, maladaptive tubular cells — particularly in the proximal tubule — arise approximately 2 days after IRI, continue to accumulate until 14 days, and may persist for 6 weeks after the initial insult (8, 17). Maladaptive cells exhibit an increasing interaction with inflammatory cells and fibroblasts and correlate with the AKI-to-CKD transition (8, 12, 18, 19). Our group recently demonstrated that failure to repair the proximal tubule is associated with a second wave of inflammatory response at 14 days after IRI, characterized by the influx of macrophages, neutrophils, and T cells, which could directly mediate renal fibrosis and kidney atrophy (15). Despite these insights regarding AKI-to-CKD transition in murine models of AKI, it is unknown how maladaptive repair early after injury can impact patients with AKI, since serial biopsies are almost never performed in patients. Previous studies, including studies from the ASSESS-AKI cohort, established the association of cross-sectional measurement of noninvasive biomarkers of kidney injury, inflammation, and tubular health with AKI. At 3 months after AKI, the increase of biomarkers of inflammation, such as urine MCP-1 and YKL-40, was associated with higher risk of AKI-to-CKD transition (14). However, most biomarker studies of AKI included measurements at a single time point in the early phase (typically less than 3 months) of AKI. Therefore, these measurements did not reflect the evolution of these biological processes over time and did not account for the later stage of AKI-to-CKD transition. In this study, we measured biomarkers at multiple time points to identify their longitudinal changes through 1 year after AKI and to provide insights regarding the etiological relationship between sustained kidney injury and inflammation as well as lack of restoration of tubular health with kidney disease progression in a large cohort of patients with AKI.

To gain mechanistic insights into the association between the longitudinal changes in kidney injury and inflammation with AKI-to-CKD transition, we performed transcriptomic analyses using mouse models of atrophy and repair after IRI, as previously reported (11, 14, 20, 21). Maladaptation and kidney atrophy after injury is associated with faster initial upregulation and higher expression of inflammation markers (*Ccl2*, *Il18*, *Tnfrsf1a*, *Tnfrsf1b*, and *Chi3l1*) even through 90 days after initial IRI. The persistent inflammation is accompanied by slower recovery of tubular health, as reflected by *Umod* expression, and slower resolution of the injury marker *Havcr1*. The persistently high expression of injury marker *Lcn2* in the early course (days 1–14) after IRI in the atrophy model is likely due to the accumulation of *Lcn2*-expressing neutrophils. These results harmonize well with our observation in the hospitalized patients. While mild injury to the kidney is associated with rapid adaptive repair and minimal progression to maladaptation, severe injury may lead to progressive accumulation of maladaptive tubular cells, particularly in the proximal tubule, and made lead to the accumulation of immune cells that persistently express these injury and inflammation markers. Our results from the mouse models of kidney atrophy and repair after IRI demonstrate that this maladaptive response may persist through 90 days after IRI, suggesting the need for further mechanistic investigation beyond the 7–14 day time point after AKI for therapeutic development to prevent long-term AKI-to-CKD transition.

Consistent with the findings in our mouse models, participants with biomarker evidence of worsening kidney injury and inflammation in the first 12 months after AKI exhibited a higher risk of CKD than their peers with resolving injury and inflammation. This association is particularly strong in the first 3 months after hospitalization with AKI. The worsening intrinsic kidney injury may be underappreciated due to its lack of correlation with changes in serum creatinine concentration. Clinicians often determine AKI recovery based on the downtrend of serum creatinine concentration after its peak. However, in mice after IRI, serum creatinine and blood urea nitrogen concentrations typically start to decrease after the first 1–2 days, while intrinsic injury and inflammation continues to worsen over 2 weeks (18). In humans, there is a similar lack of correlation between serum creatinine and kidney injury biomarkers that reflect the severity of acute tubular injury (22). The disconnect between serum creatinine improvement and progression of intrinsic injury and inflammation at the tissue level could be due to the enormous compensatory capacity from healthy and repaired nephrons. In hospitalized patients, the discrepancy between serum creatine and tissue injury and inflammation could additionally be contributed by the hemodilution effect from fluid resuscitation and the decrease in creatinine generation from the loss of muscle mass in acute illness, both of which do not necessarily reflect a change of kidney function.

The lack of recognition of worsening intrinsic injury by traditional measures (serum creatinine) may result in suboptimal patient care that contributes to long-term kidney function decline in the outpatient setting and, eventually, the development of CKD (23). For instance, potential nephrotoxic drugs may be inappropriately continued. Certain disease entities, such as acute interstitial nephritis, may be underrecognized and eventually left untreated (24). Preexisting comorbidities may additionally limit kidneys’ repair potential and further contribute to persistent or worsening injury. Transcriptomic studies in preclinical models and patients with CKD suggest that the maladaptive process, characterized by the loss of differentiated states and enrichment of the inflammatory and fibrotic milieu, may occur in the renal tubule in the setting of diabetes and aging without acute insult (13, 25). Biomarkers of tubular injury (e.g., plasma KIM-1) and inflammation (e.g., plasma TNFR1 and TNFR2) are associated with higher risks of CKD progression in patients with diabetic CKD and APOL-1–associated kidney diseases (26–29). These findings and our results suggest the possibility of AKI and CKD as a continuum in the disease spectrum at the tubular level. The renal tubular injury and inflammation at the tissue level can be quantified by longitudinally monitoring the noninvasive markers of tubular injury and tubular health.

In this study, we measured noninvasive biomarkers of kidney injury, inflammation, and tubular health at multiple time points after hospitalization in a large prospective cohort of patients with AKI. We used linear mixed effect models to account for the absence of sample collection during follow-up and conducted extensive subgroup and sensitivity analyses to ensure the validity of our findings, including the exclusion of recurrent AKI as an important confounder. The findings from preclinical AKI models and the prospective cohort of patients with AKI have several clinical implications. Longitudinal monitoring of kidney injury and inflammation may differentiate phenotypes of AKI (adaptive versus maladaptive repair) and provide prognostic value regarding complications of AKI beyond clinical parameters. Future studies could determine the optimal combination of biomarkers that could provide the best predictive performance of future CKD events for clinical implementation. Changes in serum creatinine concentration do not correlate well with the histological and transcriptional changes at the renal tubule level (22). Our results support the feasibility of using these sensitive biomarkers as potential pharmacodynamic endpoints in early phase investigations of drugs promoting kidney adaptive repair and preventing the AKI-to-CKD transition. In addition, this study highlights the importance of long-term follow-up of patients with AKI beyond the initial hospitalization course in clinical practice as well as in clinical trials of therapies aiming at mitigating long-term complications after AKI.

We recognize several limitations worth considering. Our study did not account for different etiologies of AKI to determine whether there are distinctive disease categories, such as acute interstitial nephritis, sepsis-associated AKI, and toxic tubular injury from medications, that contribute to higher risk of worsening tubular injury or inflammation. Since study participants did not undergo kidney biopsy for tissue interrogation, we could not determine whether participants with worsening injury have any histological features suggestive of maladaptation. This may be addressed by large scale tissue interrogation studies of hospitalized patients with AKI, such as the Kidney Precision Medicine Project (30). We only included participants who developed clinical AKI during hospitalization. The study of participants without AKI from the ASSESS-AKI cohort may help in characterizing the longitudinal change of subclinical tubular injury and inflammation after hospitalization for acute illness. However, this subclinical injury may be caused by chronic conditions such as aging, diabetes, and cardiovascular disease, rather than acute insults, and there are currently no established cutoff values of these biomarkers to distinguish between them. Last, our reverse translational investigations of mouse models of AKI focused on differences in the repair course (adaptive versus maladaptive repair) after IRI but did not explore the correlation between the severity of tubular injury and the repair course. Future mechanistic studies could determine whether prolonged IRI is associated with higher risk of persistent injury, inflammation, lack of restoration of tubular health, and subsequent kidney fibrosis and atrophy.

In conclusion, longitudinally monitoring biomarkers of kidney injury, inflammation, and tubular health may help characterize phenotypes of AKI. Worsening of intrinsic kidney injury and inflammation over the course of 12 months after AKI is associated with high risks of kidney disease progression.

## Methods

### Study population for patients hospitalized with AKI.

The ASSESS-AKI study is a prospective cohort study composed of 1,538 hospitalized adults with and without AKI (1:1 matched) enrolled between December 2009 and February 2015 from 4 North American clinical centers involving various hospital settings (Supplemental Table 5) (31).

Details of study design have been previously described in detail (31, 32). Briefly, 769 participants who developed AKI were recruited, and a participants in a control group who did not develop AKI were matched in 1:1 ratio based on preadmission CKD status and an integrated score including age, history of cardiovascular disease, diabetes mellitus, baseline eGFR, and treatment in an ICU. AKI was defined as an increase of serum creatinine concentration of 0.3 mg/dL or more, or at least 50% from the nearest serum creatinine (SCr) value obtained from an outpatient, nonemergency department setting within 365 days prior to hospitalization (baseline serum creatinine). Participants had their first outpatient research study visits 3 months after discharge. Follow-up study visits were conducted annually thereafter, with telephone contacts conducted at 6-month intervals. During follow-up study visits, we reviewed records from subsequent hospitalizations and used the same definition to capture recurrent AKI episodes.

In this study, we limited the analysis to participants with AKI at the initial hospitalization. As we aimed to determine the association between biomarker changes from AKI diagnosis to 12 months after discharge with kidney disease progression, the time origin of our primary survival analysis was set at 12 months after discharge, when the last exposures (biomarkers) were measured. Therefore, we excluded participants who developed incident CKD or CKD progression because they already developed the outcome prior to time origin (left censoring). We also excluded participants who did not develop the study outcome but died before their 12- month visits. We also excluded participants with missing sample collection at hospitalization and participants with missing sample collection at both 3- and 12-month visits.

### Biomarker measurement.

To characterize the longitudinal changes in biomarkers of kidney injury and inflammation in patients with AKI, we measured a panel of noninvasive biomarkers reflecting these processes at 3 time points after AKI diagnosis (hospitalization and 3 and 12 months after discharge). These biomarkers included biomarkers of kidney injury (urine albumin, KIM-1, and NGAL), inflammation (urine IL-18, MCP-1, YKL-40, and plasma TNFR1 and TNFR2), and tubular health (urine UMOD). Samples were collected within 48–96 hours of diagnosis of AKI during hospitalization and were collected in the morning of follow-up 3- and 12-month visits. After collection, samples were placed on ice if they were not processed within 30 minutes. Samples could be processed up to 6 hours after collection, were spun for 10 minutes at 1,000*g* at room temperature, aliquoted, frozen, and stored at –70°C until measurement (33). Assays for these measurements are listed in Supplemental Table 2 (33).

### Study outcome.

The study composite outcome was incident CKD or CKD progression (31). We defined incident CKD as ≥ 25% reduction in estimated GFR (eGFR) compared with baseline and achieving eGFR < 60 mL/min/1.73 m^2^ among participants without preexisting CKD (i.e., baseline eGFR ≥ 60 mL/min/1.73 m^2^). In participants with preexisting CKD, we defined CKD progression as ≥ 50% reduction in eGFR compared with baseline, an eGFR < 15 mL/min/1.73 m^2^, or receiving kidney replacement therapy or kidney transplant. We calculated eGFR using the Chronic Kidney Disease Epidemiology Collaboration 2012 equation (34).

### Animal surgery and experimental protocol.

We compared 2 mouse models of IRI (repair and atrophy) that we previously reported, and we examined the gene expression kinetics of 8 biomarkers whose longitudinal slopes were associated with AKI-to-CKD transition (14, 15). All animal protocols were approved by the Yale University Animal Care and Use Committee (IACUC protocol no. 10538). C57BL/6 (Envigo) WT male mice (age 9–11 weeks) were used in this work. All mice were maintained on a 12-hour light and 12-hour dark cycle with free access to standard food and water before and after surgery. Before surgery, all mice were subjected to anesthesia by i.p. injection with ketamine (100 mg/kg) (KETASET CIII, Zoetis) and xylazine (10 mg/kg) (AnaSed, AKORN) on a 37°C warming pad (Gaymar Stryker T/Pump System). To establish the atrophy (unilateral IRI) model, the abdomen was opened, and warm renal ischemia was induced using a nontraumatic microaneurysm clip (FST Micro Clamps) on the left renal pedicle for 27 minutes, leaving the right kidney intact. To establish the repair (unilateral IRI with contralateral nephrectomy) model, the right kidney was surgically removed at the time of left kidney ischemia. During surgery, all mice received i.p. PBS (Corning) and buprenorphine (0.1 mg/kg) (PAR Pharmaceutical) to avoid dehydration and postoperative pain, respectively. The mice were sacrificed on days 1, 7, 14, 30, and 90 after surgery (*n* = 10 mice/time point). Baseline control mice were sacrificed and denoted as day 0 for the injury (*n* = 10 mice).

### scRNA-Seq data analysis.

The scRNA-Seq data library was generated from mice kidneys from the repair model and the atrophy model on days 0 (control), 7, 14, and 30 (*n* = 2 per time point for each model). The library generation, data preprocessing, and analysis were described in detail in a recent study (15). The gene expressions of biomarkers of injury, inflammation, and tubular health were visualized across each time point in the 2 models.

### Quantitative PCR analysis.

Whole kidney RNA was extracted with an RNeasy Mini kit (Qiagen) and reverse transcribed using the iScript cDNA Synthesis Kit (Bio-Rad). Gene expression analysis was determined by quantitative PCR (qPCR) using an iCycler iQ (Bio-Rad) and normalized to hypoxanthine-guanine phosphoribosyltransferase (*Hprt*). Primers used include previously published primers for *Havcr1*, *Ccl2*, *Chi3l1*, *Umod*, and *Hprt* (14, 15, 35) as well as *Lcn2* forward: 5′-GCGGTCCAGAAAAAAACAGA-3′, reverse: 5′-GCATATTTCCCAGAGTGAAC-3′; *Il18* forward: 5′-ATTGATCAAAGTGCCAGTGAAC-3′, reverse: 5′-TGTTCTTACAGGAGAGGGTAGA-3′; *Tnfrsf1a* forward: 5′-CAAAGAGGAGAAGGCTGGAAA-3′, reverse: 5′-GGTACTGGAGACAGGAGAACTA-3′; and *Tnfrsf1b* forward: 5′-CCTCCTGGCCAATATGTGAAA-3′, reverse: 5′-ACACTCGGTTCTGCTGTTTAG-3′. The data were expressed using the ΔCt method, and the mRNA ratios were given by 2^–ΔCt^. The mRNA expression kinetics of *Havcr1* and *Ccl2* were analyzed by combining the original data from day 90 and the published data from days 0–30 (14, 15). The mRNA expression kinetics of *Chi3l1* and *Umod* were analyzed by combining the original data from day 90 and the repeated data from the newly extracted RNA with reversely transcribed cDNA from the same kidney lysates obtained from a previous study (14).

### Data availability.

The scRNA-Seq data used in this study have been deposited and are publicly available in the GEO database under accession no. GSE197626.

### Statistics.

For analyses in ASSESS-AKI participants, we converted biomarkers to their log_2_ scale. We then characterized the longitudinal monthly changes (i.e., slopes) of biomarkers of kidney injury, inflammation, and tubular health from hospitalization to 12 months after discharge. Since each participant had 2–3 biomarker measurements, we constructed linear mixed-effect models with the change of log_2_ biomarker at visits 3 months and 12 months after hospitalization as the dependent variable, and time in months as the independent variable, using maximum likelihood estimation of the random slopes. In these models, the biomarker slopes represented monthly biomarker changes from hospitalization to 12 months after discharge on the log_2_ scale. We converted the biomarker slopes to monthly percentage change for the interpretation and characterization of their distributions.

To determine the associations between biomarker slopes with the outcome, we considered the 12-month visit after initial AKI hospitalization as the time origin for study cohort. Participants who died beyond 12 months after discharge were censored. We scaled the biomarker slopes (i.e., monthly biomarker changes on their log_2_ scale) by their SD so the associations with the composite CKD outcome were comparable across biomarkers (Supplemental Table 3). We used Cox proportional hazard regression models with sequential adjustment for the following confounders: (a) adjusted for biomarker at hospitalization; (b) additionally adjusted for age, sex, self-identified race, and Hispanic ethnicity; (c) additionally adjusted for hypertension, diabetes, atherosclerotic cardiovascular disease, congestive heart failure, smoking status, baseline eGFR prior to hospitalization, urine creatinine concentration at hospitalization, and the slope of urine creatinine concentration from hospitalization to 12 months after discharge; and (d) additionally adjusted for albuminuria at hospitalization. We considered model 4 as the final adjusted model. We conducted subgroup analysis for the composite CKD outcome in participants who did not experience recurrent AKI from hospitalization to 12 months after discharge. We also performed subgroup analysis for incident CKD in participants without preexisting CKD, and for CKD progression in participants with baseline CKD. In addition, we converted biomarker slopes with tertiles, and we determined the associations with the CKD outcome using the final adjusted Cox model. In addition, we used linear mixed-effect models to determine the associations with biomarker slopes with change of eGFR from baseline, adjusting for the same sets of confounders without baseline eGFR itself.

We evaluated nonlinear relationship between biomarker slopes with the CKD outcome using restricted cubic splines with 3 knots (i.e., 33th, 67th, and 100th percentile) of biomarker slopes in the final adjusted Cox model. We visualized the distribution of hazards ratios by comparing the risk of CKD in participants with different biomarker slopes against participants with the mean biomarker slopes. We conducted a sensitivity analysis by modeling the rates of biomarker change (i.e., difference in log_2_ biomarker values from 2 time points divided by number of months) from hospitalization to 3 months after discharge and from 3 months to 12 months after discharge as 2 independent variables in the final adjusted Cox model. For all survival analyses, proportional hazard assumptions were examined by ensuring no correlation between the Schoenfeld residual and time. We considered *P* < 0.05 as statistically significant and performed all analyses using R 4.1.3.

For mouse qPCR analyses, the data were expressed as means ± SD. Two-group time course comparison was performed by 2-way ANOVA for model comparison to test whether there is a difference between the models and in the time course, followed by Bonferroni post hoc tests. All the statistical analysis was performed using Prism 8 (GraphPad Software). *P* < 0.05 was considered statistically significant.

### Study approval.

The study was approved by IRBs in the participating sites. Written informed consent was obtained from participants. All animal protocols were approved by the Yale University Animal Care and Use Committee (IACUC protocol no. 10538).

## Author contributions

Conceptualization was contributed by YW, LX, LGC, and CRP. Methodology was contributed by YW, LX, HTP, LGC, and CRP. Investigation was contributed by YW, LX, IM, HTP, LGC, and CRP. Funding acquisition was contributed by CRP. Supervision was contributed by DGM, SGC, CYH, ASG, KDL, EDS, TAI, VMC, JSK, PLK, JH, LGC, and CRP. Writing of the original draft was contributed by YW, LX, LGC, and CRP. Review and editing of the manuscript were contributed by YW, LX, HTP, DGM, SGC, CYH, ASG, KDL, EDS, TAI, VMC, JSK, PLK, JH, LGC, and CRP. Members from the ASSESS-AKI consortium contributed to cohort design and study participant enrollment.

## Supplementary Material

Supplemental data

ICMJE disclosure forms

## Figures and Tables

**Table 1:**
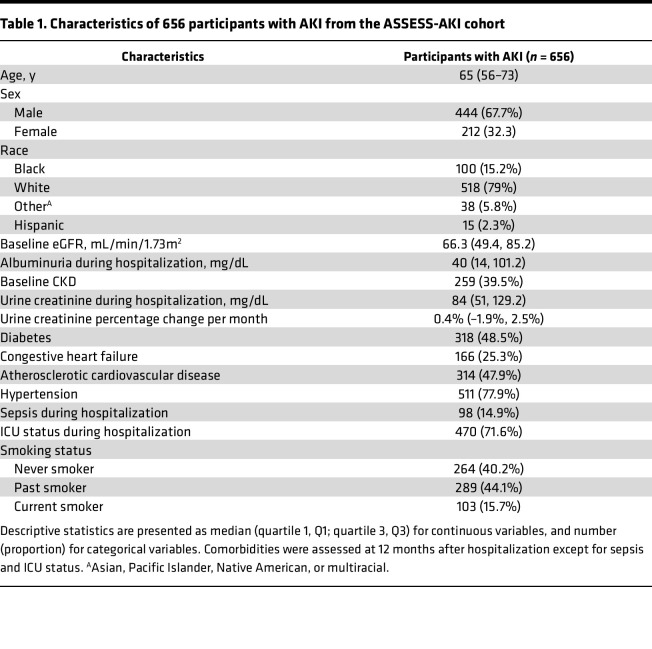
Characteristics of 656 participants with AKI from the ASSESS-AKI cohort

**Table 2:**
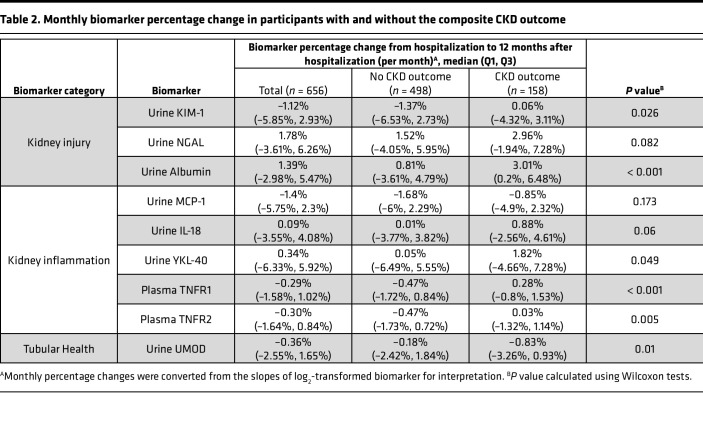
Monthly biomarker percentage change in participants with and without the composite CKD outcome

**Table 3:**
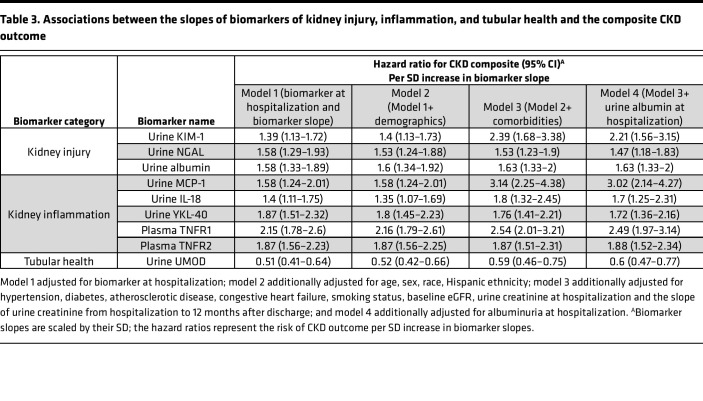
Associations between the slopes of biomarkers of kidney injury, inflammation, and tubular health and the composite CKD outcome

## Version 1 03/23/2023

In-Press Preview

## Version 2 05/08/2023

Electronic publication

## References

[1] Hsu CY (2007). Community-based incidence of acute renal failure. Kidney Int.

[2] Ronco C (2019). Acute kidney injury. Lancet.

[3] Conway BR (2020). Kidney single-cell atlas reveals myeloid heterogeneity in progression and regression of kidney disease. J Am Soc Nephrol.

[4] Chawla LS (2014). Acute kidney injury and chronic kidney disease as interconnected syndromes. N Engl J Med.

[5] See EJ (2019). Long-term risk of adverse outcomes after acute kidney injury: a systematic review and meta-analysis of cohort studies using consensus definitions of exposure. Kidney Int.

[6] Coca SG (2009). Long-term risk of mortality and other adverse outcomes after acute kidney injury: a systematic review and meta-analysis. Am J Kidney Dis.

[7] https://www.niddk.nih.gov/about-niddk/strategic-plans-reports/usrds.

[8] Kirita Y (2020). Cell profiling of mouse acute kidney injury reveals conserved cellular responses to injury. Proc Natl Acad Sci U S A.

[9] Dixon EE (2022). Spatially resolved transcriptomic analysis of acute kidney injury in a female murine model. J Am Soc Nephrol.

[10] Gerhardt LMS (2021). Single-nuclear transcriptomics reveals diversity of proximal tubule cell states in a dynamic response to acute kidney injury. Proc Natl Acad Sci U S A.

[11] Yang L (2010). Epithelial cell cycle arrest in G2/M mediates kidney fibrosis after injury. Nat Med.

[12] Ide S (2021). Ferroptotic stress promotes the accumulation of pro-inflammatory proximal tubular cells in maladaptive renal repair. Elife.

[14] Puthumana J (2021). Biomarkers of inflammation and repair in kidney disease progression. J Clin Invest.

[15] Xu L (2022). Immune-mediated tubule atrophy promotes acute kidney injury to chronic kidney disease transition. Nat Commun.

[16] He L (2017). AKI on CKD: heightened injury, suppressed repair, and the underlying mechanisms. Kidney Int.

[17] Ko GJ (2010). Transcriptional analysis of kidneys during repair from AKI reveals possible roles for NGAL and KIM-1 as biomarkers of AKI-to-CKD transition. Am J Physiol Renal Physiol.

[18] Yang L (2010). Epithelial cell cycle arrest in G2/M mediates kidney fibrosis after injury. Nat Med.

[19] Dong Y (2019). Ischemic duration and frequency determines AKI-to-CKD progression monitored by dynamic changes of tubular biomarkers in IRI mice. Front Physiol.

[20] Finn WF (1984). Attenuation of injury due to unilateral renal ischemia: delayed effects of contralateral nephrectomy. J Lab Clin Med.

[21] Fu Y (2018). Rodent models of AKI-CKD transition. Am J Physiol Renal Physiol.

[22] Moledina DG (2017). Performance of serum creatinine and kidney injury biomarkers for diagnosing histologic acute tubular injury. Am J Kidney Dis.

[23] James MT (2019). Incidence and prognosis of acute kidney diseases and disorders using an integrated approach to laboratory measurements in a universal health care system. JAMA Netw Open.

[24] Moledina DG, Perazella MA (2021). The challenges of acute interstitial nephritis: time to standardize. Kidney360.

[25] Dhillon P (2021). The nuclear receptor ESRRA protects from kidney disease by coupling metabolism and differentiation. Cell Metab.

[26] Chen TK (2022). Longitudinal TNFR1 and TNFR2 and kidney outcomes: results from AASK and VA NEPHRON-D. J Am Soc Nephrol.

[27] Schmidt IM (2022). Circulating plasma biomarkers in biopsy-confirmed kidney disease. Clin J Am Soc Nephrol.

[28] Nadkarni GN (2018). Plasma biomarkers are associated with renal outcomes in individuals with APOL1 risk variants. Kidney Int.

[29] Coca SG (2017). Plasma biomarkers and kidney function decline in early and established diabetic kidney disease. J Am Soc Nephrol.

[30] https://www.niddk.nih.gov/research-funding/research-programs/kidney-precision-medicine-project-kpmp.

[31] Hsu C-Y (2020). Post-acute kidney injury proteinuria and subsequent kidney disease progression: the assessment, serial evaluation, and subsequent sequelae in acute kidney injury ASSESS-AKI study. JAMA Intern Med.

[32] Go AS (2010). The assessment, serial evaluation, and subsequent sequelae of acute kidney injury ASSESS-AKI study: design and methods. BMC Nephrol.

[33] Liu KD (2016). Storage time and urine biomarker levels in the ASSESS-AKI study. PLoS One.

[34] Levey AS (2009). A new equation to estimate glomerular filtration rate. Ann Intern Med.

[35] Xu L (2019). Tubular GM-CSF promotes late MCP-1/CCR2-mediated fibrosis and inflammation after ischemia/reperfusion injury. J Am Soc Nephrol.

