# Supplementary material for: Longitudinal biomarkers and kidney disease progression after acute kidney injury
Source: JCI Insight. 2023 May 8;8(9):e167731. doi: 10.1172/jci.insight.167731 (PMC10243801; doi:10.1172/jci.insight.167731)
Supplement: Supplemental data [file jciinsight-8-167731-s153.pdf]

List of Supplemental Materials

Figure S1. Associations between slopes of biomarkers of kidney injury, inflammation and tubular health with the composite CKD outcome in participants without recurrent AKI

Figure S2. Associations between tertiles of the slopes of biomarkers of kidney injury, inflammation and tubular health and kidney function decline

Figure S3. Associations between slopes of biomarkers of kidney injury, inflammation and tubular health with incident CKD and with CKD progression

Figure S4. Associations between biomarker slopes from hospitalization to 3- months visits, and from 3- to 12-months visits with the composite CKD outcome

Table S1. Monthly biomarker percentage change in participants without recurrent AKI

Table S2. Biomarker measurement assays

Table S3. Mean and standard deviation of biomarker slopes

Table S4. Significance levels comparing biomarker gene expression in kidneys from mouse models of atrophy and repair after ischemic reperfusion injury

Table S5. STROBE checklist.

Supplemental Figure S1. **Associations between slopes of biomarkers of kidney injury, inflammation and tubular health with the composite CKD outcome in participants without recurrent AKI**

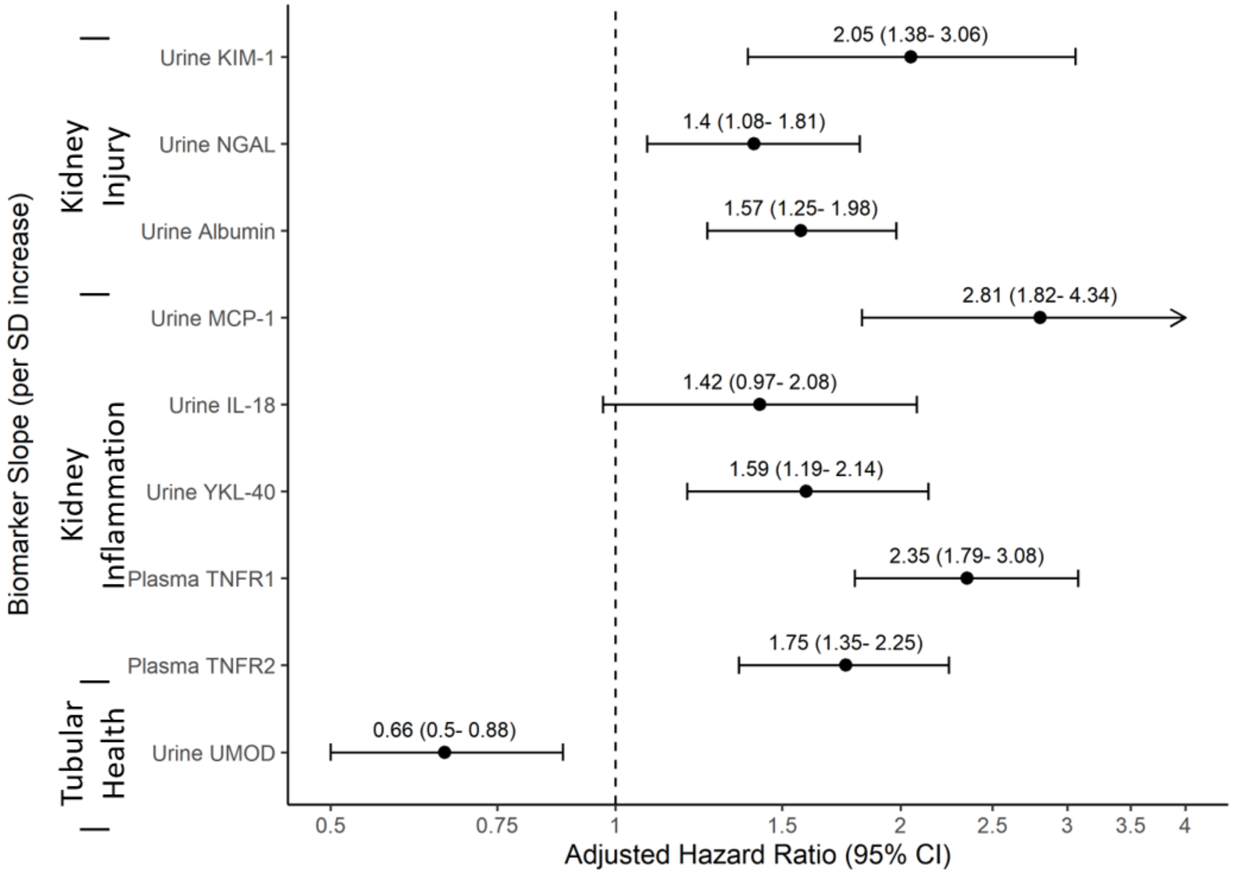

Five hundred and sixty-one participants did not have recurrent AKI from hospitalization to 12 months after discharge.

Model adjusted for biomarker at hospitalization, age, sex, self-identified race, Hispanic ethnicity, hypertension, diabetes, atherosclerotic cardiovascular disease, congestive heart failure, smoking status, baseline eGFR prior to hospitalization, albuminuria at hospitalization, urine creatinine at hospitalization and the slope of urine creatinine from hospitalization to 12 months after discharge.

Supplemental Figure S2. Associations between tertiles of the slopes of biomarkers of kidney injury, inflammation and tubular health and kidney function decline

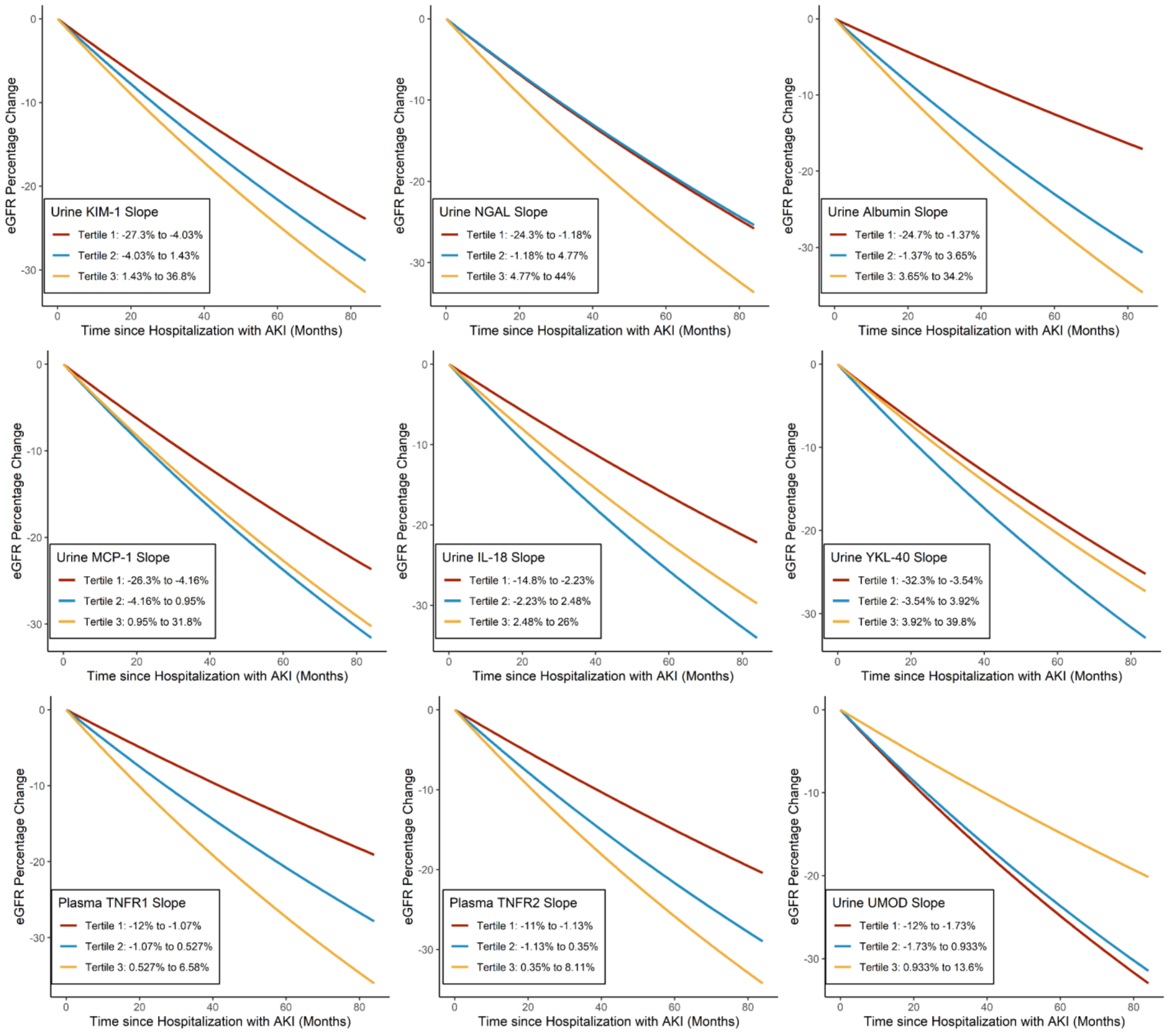

Supplemental Figure S3. **Associations between slopes of biomarkers of kidney injury, inflammation and tubular health with incident CKD and with CKD progression**

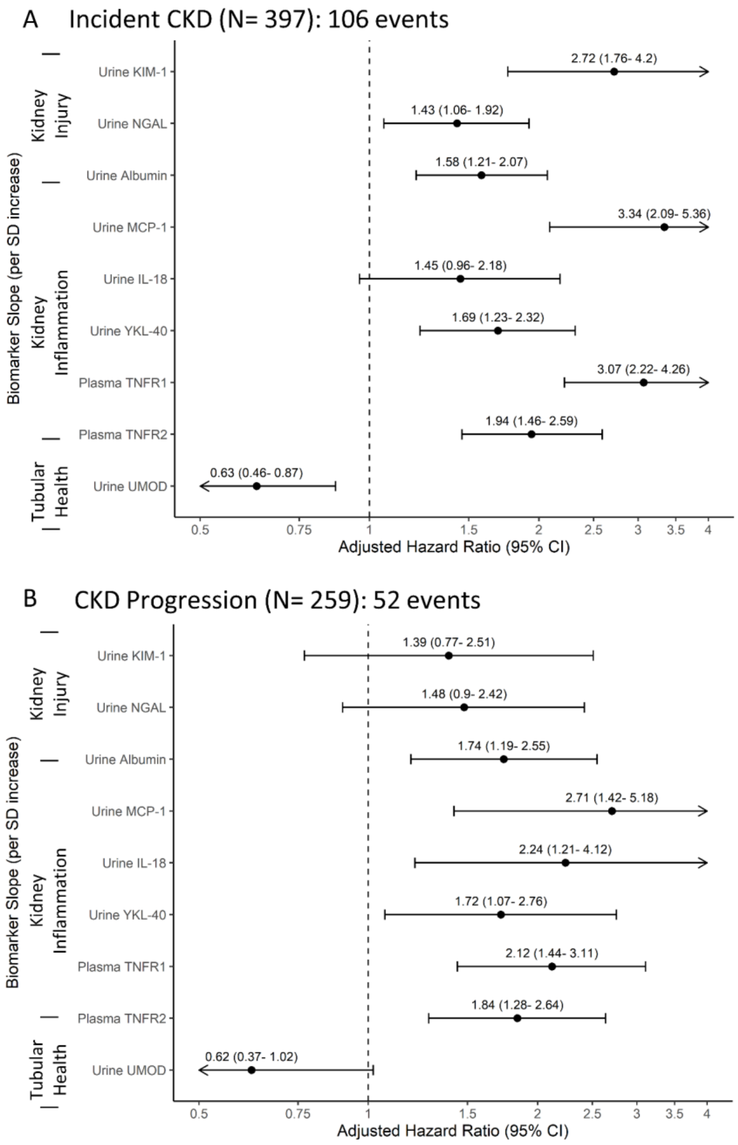

(A) Biomarker slopes' associations with incident CKD in 397 participants without pre-existing CKD; (B) Biomarker slopes' associations with CKD progression in 259 participants with pre-existing CKD.

Model adjusted for biomarker at hospitalization, age, sex, self-identified race, Hispanic ethnicity, hypertension, diabetes, atherosclerotic cardiovascular disease, congestive heart failure, smoking status, baseline eGFR prior to hospitalization, albuminuria at hospitalization, urine creatinine at hospitalization and the slope of urine creatinine from hospitalization to 12 months after discharge.

Supplemental Figure S4. Associations between biomarker slopes from hospitalization to 3- months visits, and from 3- to 12-months visits with the composite CKD outcome

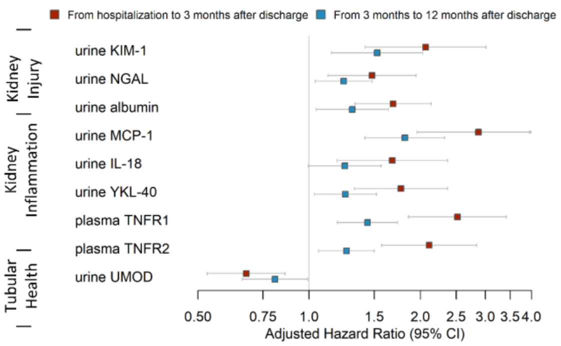

Hazard Ratio for biomarker slope from hospitalization to 3 months after discharge	Hazard Ratio for biomarker slope from 3 to 12 months after discharge
2.07 (1.42- 3.01)	1.53 (1.15- 2.03)
1.48 (1.12- 1.95)	1.24 (1.04- 1.48)
1.68 (1.19- 2.37)	1.25 (1- 1.57)
2.87 (1.96- 4.2)	1.82 (1.42- 2.33)
1.69 (1.33- 2.14)	1.31 (1.04- 1.64)
1.77 (1.33- 2.37)	1.25 (1.03- 1.52)
2.52 (1.86- 3.42)	1.44 (1.19- 1.74)
2.11 (1.57- 2.84)	1.26 (1.06- 1.5)
0.68 (0.53- 0.86)	0.81 (0.66- 0.99)

Five hundred and eighty-one participants had urine biomarkers measured at all three timepoints, and 541 participants had plasma biomarkers measured at all three timepoints.

Biomarker slopes at both time intervals were scaled by their standard deviations, so the hazard ratios represent the risk of CKD outcome per standard deviation increase in biomarker slopes.

Model adjusted for biomarker at hospitalization, age, sex, self-identified race, Hispanic ethnicity, hypertension, diabetes, atherosclerotic cardiovascular disease, congestive heart failure, smoking status, baseline eGFR prior to hospitalization, albuminuria at hospitalization, urine creatinine at hospitalization and the slope of urine creatinine from hospitalization to 12 months after discharge.

Supplemental Table S1. Monthly biomarker percentage change in participants without recurrent AKI

Biomarker Category	Biomarker	Biomarker Percentage Change from hospitalization to 12 months after hospitalization (per month)*, median (Q1, Q3)			
		Total (N=561)	No CKD Outcome (N=440)	CKD Outcome (N=121)	p Value#
Kidney Injury	Urine KIM-1	-1.15% (-6.08%, 2.86%)	-1.43% (-6.59%, 2.65%)	-0.69% (-4.94%, 3.49%)	0.08
	Urine NGAL	1.47% (-3.94%, 6.13%)	1.11% (-4.42%, 5.89%)	2.19% (-1.54%, 8.12%)	0.079
	Urine Albumin	1.07% (-3.22%, 5.08%)	0.76% (-3.84%, 4.66%)	3.04% (-0.82%, 6.26%)	< 0.001
Kidney Inflammation	Urine MCP-1	-1.32% (-5.8%, 2.28%)	-1.64% (-6.04, % 2.17%)	-0.81% (-5.44%, 2.33%)	0.262
	Urine IL-18	0.02% (-3.71%, 3.94%)	-0.18% (-4.13%, 3.67%)	1.02% (-2.99%, 4.99%)	0.036
	Urine YKL-40	0.1% (-6.65%, 5.87%)	-0.22% (-6.71%, 5.19%)	2.04% (-5.37%, 8.15%)	0.024
	Plasma TNFR1	-0.41% (-1.68%, 0.94%)	-0.55% (-1.76%, 0.76%)	0.17% (-1.22%, 1.38%)	0.002
	Plasma TNFR2	-0.38% (-1.72%, 0.8%)	-0.52% (-1.86%, 0.64%)	0.01% (-1.46%, 1.09%)	0.014

**Tubular Health**	**Urine UMOD**	-0.26% (-2.51%, 1.8%)	-0.11% (-2.38%, 1.94%)	-0.72% (-3.24%, 1.28%)	0.041

*Monthly percentage changes were converted from the slopes of log2-transformed biomarker for interpretation.

#p value calculated using Wilcoxon tests.

Supplemental Table S2. Biomarker measurement assays

Biomarker	Biomarker Name	Urine or Plasma	Assay
KIM-1	Kidney injury molecule-1	Urine	Duoset DY1750, R & D Systems Inc., Minneapolis, MN
IL-18	Interleukin-18	Urine	Medical & Biological Laboratories Co., Nagoya, Japan
MCP-1	Monocyte chemoattractant protein-1	Urine	Meso Scale Diagnostics, Gaithersberg, MD
UMOD	Uromodulin	Urine	Meso Scale Diagnostics, Gaithersberg, MD
NGAL	Neutrophil gelatinase-associated lipocalin	Urine	NGAL ELISA Kit 036; Bioporto, Grusbakken, Denmark
YKL-40	Chitinase 3-like 1	Urine	Meso Scale Diagnostics, Gaithersberg, MD
Albumin	Albumin	Urine	Siemens ProSpec analyzer (Siemens GMBH)
TNFR1	Soluble tumor necrosis factor receptor-1	Plasma	Meso Scale Diagnostics, Gaithersberg, MD
TNFR2	Soluble tumor necrosis factor receptor-2	Plasma	Meso Scale Diagnostics, Gaithersberg, MD

Supplemental Table S3. Mean and standard deviation of biomarker slopes

Biomarker	Biomarker Slope (log2 change per month), mean (SD)	Biomarker Monthly Percentage Change*, mean (SD)
Urine KIM-1	-0.024 (0.109)	-1.36% (7.42%)
Urine NGAL	0.017 (0.125)	1.57% (8.73%)
Urine Albumin	0.011 (0.110)	1.08% (7.53%)
Urine MCP-1	-0.030 (0.107)	-1.78% (7.21%)
Urine IL-18	0.003 (0.090)	0.38% (6.31%)
Urine YKL-40	-0.005 (0.148)	0.18% (10.24%)
Plasma TNFR1	-0.007 (0.034)	-0.44% (2.35%)
Plasma TNFR2	-0.009 (0.036)	-0.58% (2.46%)
Urine UMOD	-0.007 (0.052)	-0.45% (3.60%)

*Biomarker monthly percentage change was calculated from biomarker slope

Supplemental Table S4. Significance levels comparing biomarker gene expression in kidneys from mouse models of atrophy and repair after ischemic reperfusion injury

Biomarker gene\ Significance level	model factor (atrophy vs. repair model)	time factor	model- time interaction
Havcr1	<0.0001	<0.0001	<0.0001
Lcn2	0.4888	<0.0001	<0.0001
Ccl2	<0.0001	<0.0001	<0.0001
Il18	<0.0001	<0.0001	<0.0001
Chi3l1	<0.0001	<0.0001	<0.0001
Tnfrsf1a	<0.0001	0.0012	0.0012
Tnfrsf1b	<0.0001	<0.0001	<0.0001
Umod	0.0003	<0.0001	<0.0001

Supplemental Table S5. STROBE Statement—checklist of items that should be included in reports of observational studies

	Item No.	Recommendation	Page No.	Relevant text from manuscript
**Title and abstract**	1	(*a*) Indicate the study's design with a commonly used term in the title or the abstract	3	
		(*b*) Provide in the abstract an informative and balanced summary of what was done and what was found	3	
**Introduction**				
Background/rationale	2	Explain the scientific background and rationale for the investigation being reported	5-6	
Objectives	3	State specific objectives, including any prespecified hypotheses	6	
**Methods**				
Study design	4	Present key elements of study design early in the paper	6, 16	
Setting	5	Describe the setting, locations, and relevant dates, including periods of recruitment, exposure, follow-up, and data collection	16	
Participants	6	(*a*) *Cohort study*—Give the eligibility criteria, and the sources and methods of selection of participants. Describe methods of follow-up<br>*Case-control study*—Give the eligibility criteria, and the sources and methods of case ascertainment and control selection. Give the rationale for the choice of cases and controls<br>*Cross-sectional study*—Give the eligibility criteria, and the sources and methods of selection of participants	16	
		(*b*) *Cohort study*—For matched studies, give matching criteria and number of exposed and unexposed<br>*Case-control study*—For matched studies, give matching criteria and the number of controls per case	16	

Variables	7	Clearly define all outcomes, exposures, predictors, potential confounders, and effect modifiers. Give diagnostic criteria, if applicable	17
Data sources/ measurement	8*	For each variable of interest, give sources of data and details of methods of assessment (measurement). Describe comparability of assessment methods if there is more than one group	17
Bias	9	Describe any efforts to address potential sources of bias	16
Study size	10	Explain how the study size was arrived at	16-17

Continued on next page

Quantitative variables	11	Explain how quantitative variables were handled in the analyses. If applicable, describe which groupings were chosen and why	19-20
Statistical methods	12	(*a*) Describe all statistical methods, including those used to control for confounding	19-20
		(*b*) Describe any methods used to examine subgroups and interactions	20
		(*c*) Explain how missing data were addressed	17, 19
		(*d*) *Cohort study*—If applicable, explain how loss to follow-up was addressed<br>*Case-control study*—If applicable, explain how matching of cases and controls was addressed<br>*Cross-sectional study*—If applicable, describe analytical methods taking account of sampling strategy	20
		(*e*) Describe any sensitivity analyses	20
**Results**			
Participants	13*	(a) Report numbers of individuals at each stage of study—eg numbers potentially eligible, examined for eligibility, confirmed eligible, included in the study, completing follow-up, and analysed	6, 28
		(b) Give reasons for non-participation at each stage	6, 19
		(c) Consider use of a flow diagram	Fig 1
Descriptive data	14*	(a) Give characteristics of study participants (eg demographic, clinical, social) and information on exposures and potential confounders	6-7, Table 1
		(b) Indicate number of participants with missing data for each variable of interest	NA
		(c) *Cohort study*—Summarise follow-up time (eg, average and total amount)	7
Outcome data	15*	*Cohort study*—Report numbers of outcome events or summary measures over time	7
		Case-control study—Report numbers in each exposure category, or summary measures of exposure	
		Cross-sectional study—Report numbers of outcome events or summary measures	

Main results	16	(*a*) Give unadjusted estimates and, if applicable, confounder-adjusted estimates and their precision (eg, 95% confidence interval). Make clear which confounders were adjusted for and why they were included	8
		(*b*) Report category boundaries when continuous variables were categorized	29, Fig 2
		(*c*) If relevant, consider translating estimates of relative risk into absolute risk for a meaningful time period	NA

Continued on next page

Other analyses	17	Report other analyses done—eg analyses of subgroups and interactions, and sensitivity analyses	8- 9
**Discussion**			
Key results	18	Summarise key results with reference to study objectives	11
Limitations	19	Discuss limitations of the study, taking into account sources of potential bias or imprecision. Discuss both direction and magnitude of any potential bias	15
Interpretation	20	Give a cautious overall interpretation of results considering objectives, limitations, multiplicity of analyses, results from similar studies, and other relevant evidence	15
Generalisability	21	Discuss the generalisability (external validity) of the study results	13-14
**Other information**			
Funding	22	Give the source of funding and the role of the funders for the present study and, if applicable, for the original study on which the present article is based	22

*Give information separately for cases and controls in case-control studies and, if applicable, for exposed and unexposed groups in cohort and cross-sectional studies.

Note: An Explanation and Elaboration article discusses each checklist item and gives methodological background and published examples of transparent reporting. The STROBE checklist is best used in conjunction with this article (freely available on the Web sites of PLoS Medicine at http://www.plosmedicine.org/, Annals of Internal Medicine at http://www.annals.org/, and Epidemiology at http://www.epidem.com/). Information on the STROBE Initiative is available at www.strobe-statement.org

Supplemental Acknowledgments

ASSESS-AKI consortium:

Johns Hopkins University, Baltimore, MD: Chirag R. Parikh, Steven Menez, and Yumeng Wen;

Icahn School of Medicine at Mount Sinai, New York, NY: Steven G. Coca;

Yale School of Medicine, New Hanen, CT: Dennis G. Moledina;

Western University, London, Canada: Amit X. Garg;

Kaiser Permanente Northern California, Oakland, CA: Alan S. Go, Sijie Zheng and Leonid V. Pravoverov;

University of California, San Francisco, San Francisco, CA: Chi-yuan Hsu, Raymond K. Hsu, and Kathleen D. Liu;

Pennsylvania State University, Hershey, PA: Vernon M. Chinchilli, Nasrollah Ghahramani;

University of Texas San Antonio, San Antonio, TX: W. Brian Reeves;

Vanderbilt University, Nashville, TN: T. Alp Ikizler, Edward D. Siew, Julia B. Lewis and Lorraine Ware;

Cincinnati Children’s Hospital, Cincinnati, OH: Prasad Devarajan, Catherine D. Krawczeski, and Michael R. Bennett;

Montreal Children’s Hospital, Montreal, Canada: Michael Zappitelli;

University of Washington, Seattle, WA: Jonathan Himmelfarb, Mark M. Wurfel;

New York University, New York, NY: James S. Kaufman;

National Institute of Health, Bethesda, MD: Paul L. Kimmel;

University of Iowa, Iowa City, IA: John B. Stokes (in memoriam).
